# A Comprehensive Review on the Incremental Sheet Forming of Polycarbonate

**DOI:** 10.3390/polym16213098

**Published:** 2024-11-03

**Authors:** Antonio Formisano, Massimo Durante

**Affiliations:** Department of Chemical, Materials and Production Engineering, University of Naples Federico II, 80125 Napoli, Italy; mdurante@unina.it

**Keywords:** thermoplastic forming, formability, defectiveness, forming forces, surface quality, hybrid processes, numerical modelling, future perspectives

## Abstract

Incremental sheet forming has emerged as an excellent alternative to other material forming procedures, incrementally deforming flat metal sheets into complex three-dimensional profiles. The main characteristics of this process are its versatility and cost-effectiveness; additionally, it allows for greater formability compared to conventional sheet forming processes. Recently, its application has been extended to polymers and composites. The following review aims to present the current state of the art in the incremental sheet forming of polycarbonate, an outstanding engineering plastic, beginning with initial studies on the feasibility of this process for polymers. Attention is given to the advantages, drawbacks, and main applications of incrementally formed polycarbonate sheets, as well as the influence of process parameters and toolpath strategies on features such as formability, forming forces, deformation and failure mechanisms, geometric accuracy, surface quality, etc. Additionally, new hybrid forming methods for process optimisation are presented. Finally, a discussion is provided on the technical challenges and future research directions for incremental sheet forming of polycarbonate and, more generally, thermoplastics. Thus, this review aims to offer an extensive overview of the incremental forming of polycarbonate sheets, useful to both academic and industrial researchers working on this topic.

## 1. Introduction

This section provides a general introduction to incremental sheet forming, tracing its origins, followed by the current state of the art of this technology as applied to thermoplastics.

### 1.1. General Introduction to the Process

The interest in developing highly flexible procedures, such as additive manufacturing technologies, has been bolstered by recent significant advances in computer applications in manufacturing [1]. Incremental sheet forming (ISF) fits into this context. This relatively recent technology is widely used as a rapid prototyping method [2] and is cost-effective because it does not require dedicated equipment in its basic variants. It also allows for the high customisation of small-batch, non-axisymmetric sheet parts, making it suitable for potential applications in aerospace, automotives, and other fields [3,4], thanks to the layered manufacturing principle typical of rapid prototyping.

**Figure 1:**
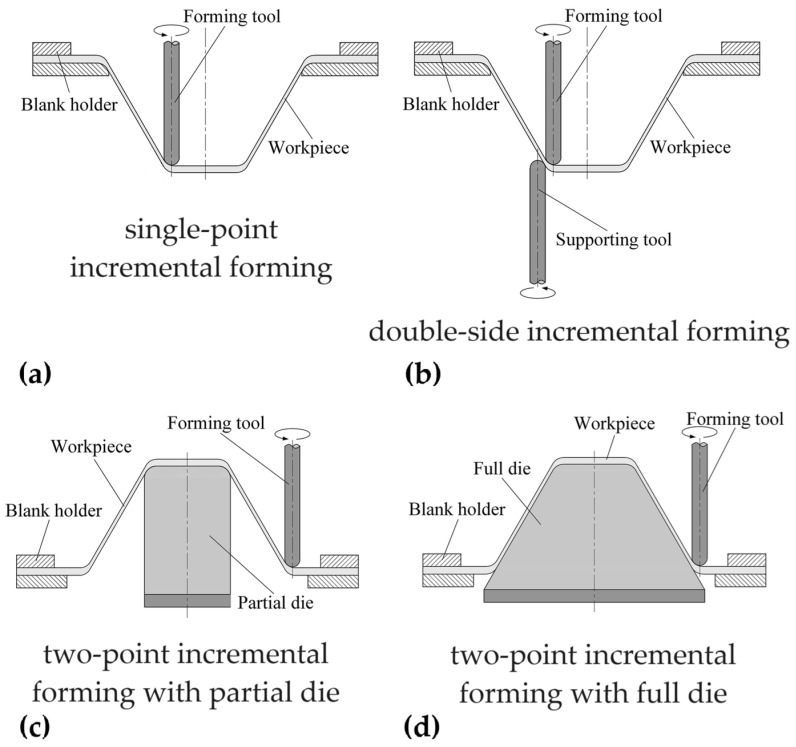
Types of incremental sheet forming processes: (**a**) single-point incremental forming (SPIF); (**b**) double-side incremental forming (DSIF); (**c**) two-point incremental forming (TPIF) with partial die and (**d**) with full die.

The principal concept of the ISF process (with various types developed; see Figure 1 for the most common ones) is the progressive deformation of a clamped sheet of pure metals, alloys, polymers, and composites [5,6] through the action of a forming tool controlled by a computerised numerical control (CNC) machine that follows a path and deforms the sheet progressively into its final shape [7].

The basic and most common version of the process is single-point incremental forming (SPIF; see Figure 1a), which uses only the forming tool and a blank holder to keep the sheet in a fixed position without additional support. In this variant, the sheet is deformed from the outside to the inside, with its centre gradually moving downwards [8].

In double-side incremental forming (DSIF; see Figure 1b), a second tool is used on the opposite side of the sheet to provide local support [9]. The sheet is then deformed under the cooperative effect of the two tools, improving deformation stability and reducing material thinning.

Two-point incremental forming (TPIF) involves using a partial (Figure 1c) or full die (Figure 1d) to support the sheet on the opposite side [10,11]; in this method, the sheet is deformed from the inside to the outside, while the flange moves downwards.

The main process parameters are the shape and dimensions of the tool, the forming temperature, and the characteristics of the toolpath (step size and forming speeds). They have been abundantly investigated and an exhaustive overview is provided in the review by Gatea et al. [12]; the correct selection of them significantly affects features such as the formability, deformation and failures, springback, accuracy, and surface quality of the formed parts.

Since its inception, research on ISF has primarily focused on its applicability to different metallic materials such as aluminium, magnesium, titanium, and their alloys. Several reviews have been published, tracing the evolution of ISF from its origin. An initial review by Jeswiet et al. [7] introduced the process from its genesis to 2005, followed by the works of Echrif and Hrairi [13], Emmens et al. [14], and Behera et al. [6], covering the period up to 2015. More recent literature reviews (up until 2023) have reported the scientific progress and future developments of ISF [15,16,17].

In addition to the general reviews mentioned above, other reviews have focused on more specific topics such as the improved formability guaranteed by ISF [18], deformation [19] and failure mechanisms [20], and analyses of the forming forces [21].

**Figure 2:**
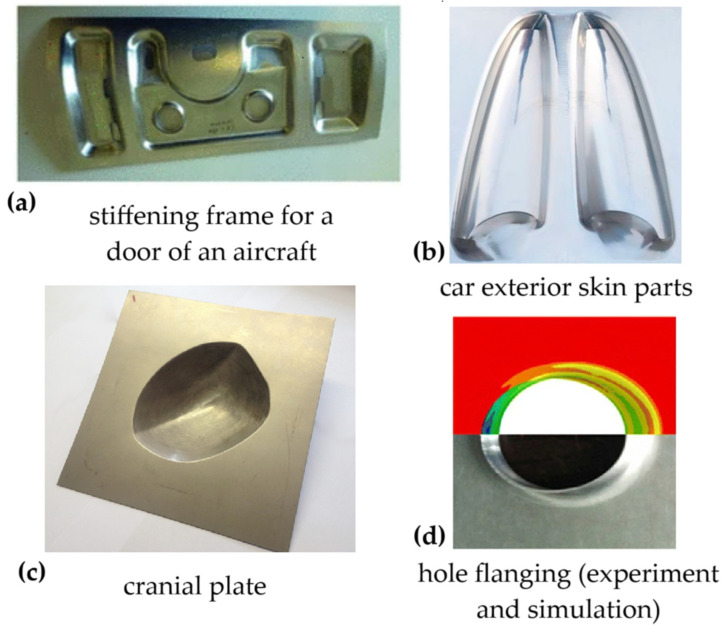
Metal parts obtained by ISF: (**a**) stiffening frame for a door of an aircraft; (**b**) car exterior skin parts; (**c**) cranial plate; (**d**) hole flanging (experiment and simulation).

The application of ISF for the manufacture of metal parts spans several industrial fields. For example, applications include the stiffening frame for a hydraulic access door of an aircraft [22], car exterior skin parts [23], and cranial plates [24], among others; additionally, a promising research direction is the formation of hole flanging by ISF [25,26]. All these parts are shown in Figure 2a–d.

### 1.2. Incremental Forming of Polymer Sheets

While early research on ISF mainly focused on metals, recent studies have shown increased interest in transferring this knowledge to hard-to-form non-metallic materials [27]. For example, consider the preliminary studies on the incremental forming of sandwich panels [28] and composite materials [29], advances in the ISF of common polymer-based composite materials using glass [30,31] and carbon fibres as reinforcement [32], and the characterisation of nanocomposites suitable for SPIF by mixing polyamide 12 and montmorillonite filler clay [33], as well as the SPIF of shape memory polymer foams [34] and thermoplastics in general.

Thermoplastics have desirable properties such as a light weight, strength, corrosion resistance, cost-effectiveness, etc., making them widely used in the manufacturing industry [35], especially for mass production. Conventional processes for these materials require repetitive heating, shaping, and cooling actions [36], leading to high costs in terms of energy and investments in equipment and tools. Thermoplastic sheet components with various shapes are frequently manufactured using common sheet metal forming processes that strongly depend on the temperature and material properties [37]. In this context, ISF can be an effective alternative to conventional technologies based on heating–shaping–cooling operations, ensuring high levels of material formability even at room temperature, as well as economic benefits [38]; moreover, some studies have highlighted relevant advantages in terms of impact resistance, temperature resistance, and bearing capacity [39,40].

Thanks to its flexibility, the ISF process is recommended for producing small and medium-sized batches in several fields [41]. For example, it can be used to manufacture aircraft canopies from polymer sheets [2] or customised medical prostheses like cranial implants using biocompatible polymers [42,43,44].

The first significant attempt to form a thermoplastic by ISF, specifically polyvinylchloride, was in 2009 [45]. Subsequent investigations explored different kinds of commercial thermoplastics, as well as new solutions and materials such as biocompatible polycaprolactone [37] and bilayer polymeric sheets [46]. These works confirmed the feasibility of the process applied to polymers.

Subsequent research analysed the influence of the main process parameters on the formability of different polymers. For example, preliminary studies on polypropylene [39] and on polyamide, polycarbonate, polyethylene terephthalate, and polyvinylchloride [47] examined the effects of forming force and temperature [48], as well as incremental depth and tool rotation [49] on the ISF of polymers.

**Figure 3:**
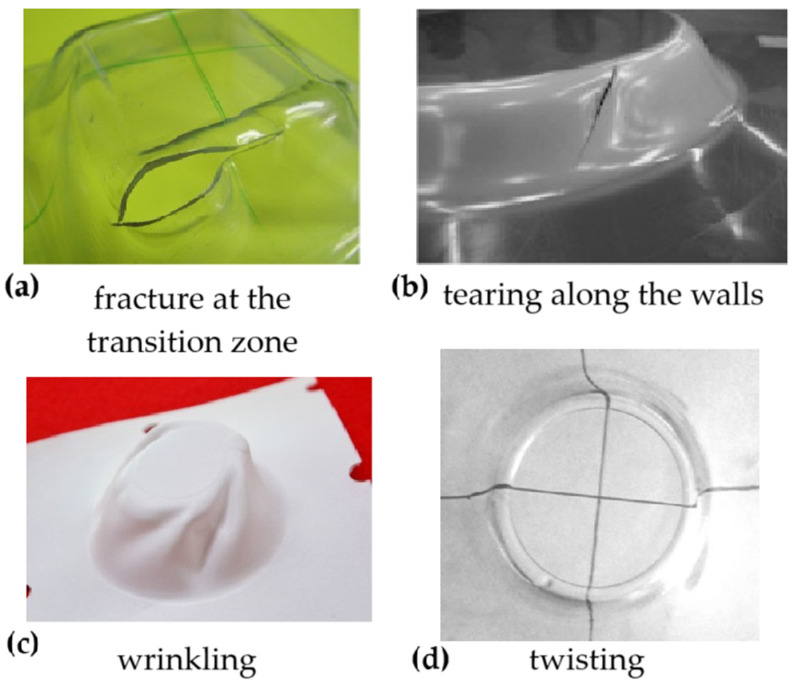
Typical failures and defects that affect ISF polymers: (**a**) fracture at the transition zone; (**b**) tearing along the walls; (**c**) wrinkling; (**d**) twisting.

Another significant field of investigation in ISF is related to failures and defects, as they influence the formability and geometrical accuracy of the formed parts [27,37]. Typical problems affecting polymer parts manufactured by ISF include ductile fracture at the transition zone between the wall and the corner radius (Figure 3a) and tearing along the walls (Figure 3b) [45,50], as well as defects like wrinkling (Figure 3c) and twisting (Figure 3d) [50,51].

These defects are strongly connected: wrinkles can twist around the axis of revolution in the direction of tool rotation. Twisting is caused by the uncontrolled pivoting of the parts around the clamping frame due to in-plane shear generated by the tangential forces applied by the forming tool on the sheet. It is more likely with higher and more regular plane forces, which cause continued strain accumulation and asymmetric strain levels [52,53]. Although twisting is a common phenomenon for all materials subject to ISF, its magnitude is particularly significant for materials like thermoplastics that exhibit soft behaviour. For this family of materials, higher normal forces can produce significant indentation that accentuates the phenomenon [54]; for example, the twist angles on axisymmetric ISF parts obtained by a unidirectional toolpath were about 6° and 22° for aluminium alloy [55] and polycarbonate sheets [50], respectively.

## 2. Incremental Forming of Polycarbonate Sheets

In this section, the main characteristics and the ISF formability of polycarbonate are reported.

### 2.1. Polycarbonate: Main Properties

Polycarbonate, also known as a “transparency metal” for its fascinating properties [56], is a lightweight and 100% recyclable amorphous thermoplastic; it is one of the most interesting polymers because it combines significant mechanical and physiochemical properties such as toughness, stiffness, strength, heat and flame resistance, high durability, shatter resistance, thermostability, good electrical insulation, and excellent transparency [57,58]. Polycarbonate is an outstanding engineering plastic used in various fields such as communication, optical/lighting, glass replacement, medical apparatus, transport, household products, aerospace, electrical and safety products, and more [59,60].

Parent polycarbonate is an isotropic elastoplastic material with mechanical behaviour that is strongly different from that of metals, as it is highly influenced by the working conditions. While metals exhibit more or less well-ordered crystalline lattices of atoms, polycarbonate consists of molecules of carbon atoms bonded into long chains, resembling a tangled collection of yarn scraps, that can rearrange into infinite different conformations depending on several parameters, such as the stress level [61]; specifically, the chain orientation is a phenomenon unique to polymers. Anisotropy emerges when molecules align along a certain direction, resulting from strong covalent bonds along the chain axis and weaker secondary bonds in the transverse direction [62].

**Figure 4:**
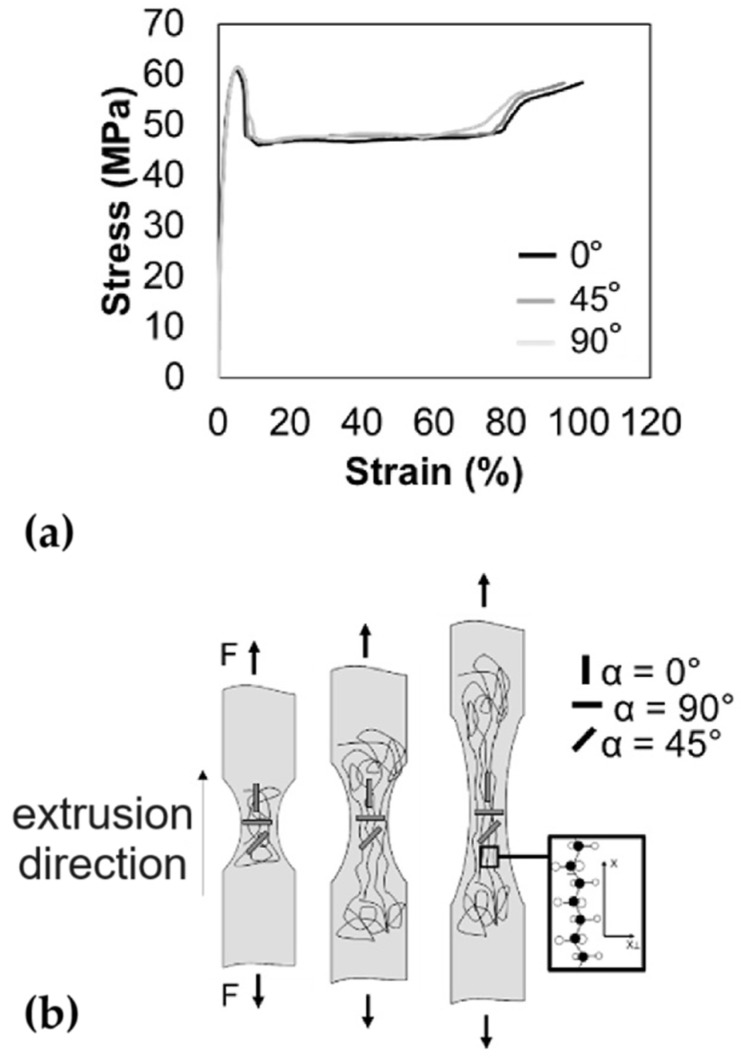
Mechanical behaviour of polycarbonate: (**a**) engineering stress–strain curves and (**b**) chain orientation of polycarbonate specimens.

For a better understanding [63], refer to the tensile tests carried out following the ASTM standard D638-14 [64]; they determine the typical engineering stress–strain curves for engineering thermoplastics below the glass transition temperature [62], as reported in Figure 4a for specimens cut at 0, 45, and 90° relative to the extrusion direction. The first linear viscoelastic region is where the polymer chains undergo stretching and disentanglement in response to applied stress. This region ends at the yield point, after which increased strain occurs with reduced stress. This phase involves the breaking of van der Waals bonds and the occurrence of permanent deformations such as necking. Subsequently, the mechanically induced orientation of polymer chains (see Figure 4b) results in a steepening slope, up to the material’s breaking point [65].

### 2.2. Formability of Polycarbonate Worked by ISF

The ISF method generates large regions of homogenous deformation, avoiding significant stress and strain gradients [66]; consequently, it guarantees a higher material formability compared to that of conventional sheet forming processes. However, some research analysing the formability of ISF polymer parts uses metal-derived methods [67], which are not always representative of the thermoplastics’ behaviour; the occurrence of failures and defects like twisting and wrinkling in polymer ISF parts greatly affects the product quality, making it necessary to consolidate established methods or develop new procedures for formability evaluation.

**Figure 5:**
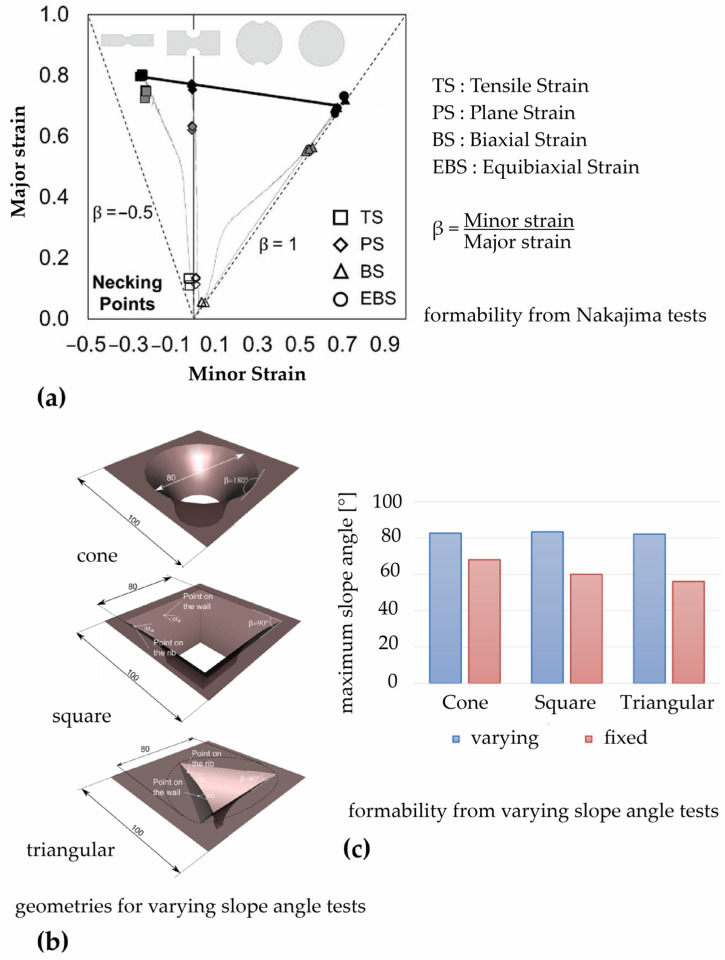
Formability of polycarbonate sheets: (**a**) forming limit curves from Nakajima tests on 2 mm thick sheets; (**b**) geometries used for varying slope angle tests and (**c**) maximum slope angle from varying and fixed slope angle tests on 1.4 mm thick sheets.

In Refs. [36,63], Nakajima specimens with different geometries were used to induce strain in tensile, plane, biaxial, and equibiaxial states through an Erichsen model 142-20 universal sheet metal testing machine, following the ISO standard 12004-2 [68], to determine the formability limits through the necking and fracture of polycarbonate sheets 1 and 2 mm in thickness. For the formability limits for necking, the time-dependent methodology for metals was used [69], while the limits for fracture were determined by measuring the principal strains through a digital image correlation (DIC) system, due to the significant springback of polymers. This methodology proved adequate for identifying the onset of necking in polycarbonate sheets; moreover, the forming limit curves described in the principal strain space were well represented by straight lines, unlike the typical V-shape seen in conventional metal forming processes (see Figure 5a).

According to the method proposed by Hussain et al. for metal sheets [66], another way to investigate the formability of polycarbonate sheets involves conducting varying slope angle tests that use a curved-line generatrix to create a revolved surface whose slope angle varies continuously, instead of creating several fixed slope angle geometries with increasing angle; they are stopped as soon as the workpiece fails, and the corresponding angle is determined. Durante et al. [51] carried out both fixed and varying slope angle tests on 1.4 and 1.9 mm thick sheets, varying the tool diameter. They investigated cone and pyramid frusta (with square and triangular bases); the latter introduced significant geometrical singularities such as sloped ribs to further stress the material’s formability. The tests highlighted the high formability of the polycarbonate sheets, regardless of the shape of the parts. The maximum slope angle increased with the tool diameter, while the thickness of the sheets showed a minor influence; the pyramid frusta recorded a high strain concentration that reduced the formability of the sheets. Moreover, the varying slope angle tests (see the corresponding geometries in Figure 5b) overestimated the formability of the sheets compared to that of the fixed ones (see Figure 5c); under the heaviest conditions (pyramid frusta with triangular base), the high discrepancy between the two tests made the varying slope angle test not fully representative of the formability of the polycarbonate sheets.

## 3. Enhancement of Incremental Forming of Polycarbonate Sheets

This section presents some solutions and tools for optimising the forming process applied to polycarbonate sheets. The reasons for this research include attempts to improve the accuracy and range of feasible geometries, as well as the formability of materials that are difficult to work with at room temperature using conventional ISF. For example, considering metal sheets, several methods of heat-assisted ISF developed to improve the formability of materials like magnesium and titanium alloys are described in a review by Liu [70]. Additionally, the development of hybrid processes for low-volume production of sheet metal parts, which combine ISF with stretch forming and laser heat treatment, as well as the forming of hybrid materials, is presented in [71].

### 3.1. Toolpath Strategy

Two studies by Formisano et al. [72,73] explore the possibility of reducing the defectiveness of polycarbonate components obtained by SPIF of 1 and 1.5 mm thick sheets by choosing a more suitable toolpath strategy; they highlight that monitoring and measuring the ISF forces represent an efficient tool for controlling process quality [74].

The studies began by observing a significant reduction in twisting for metal [7] and polycarbonate parts [50] using an alternate toolpath instead of a unidirectional one. However, this solution proved ineffective against instabilities and wrinkling on thin thermoplastic sheets [51]. The experimental campaign included the analysis of forming forces, deformation states, surface quality, failures, and defects of cone frusta with fixed wall angles, obtained using a reference and different stair-based unidirectional helical toolpath strategies, under both lubricated and dry conditions.

**Figure 6:**
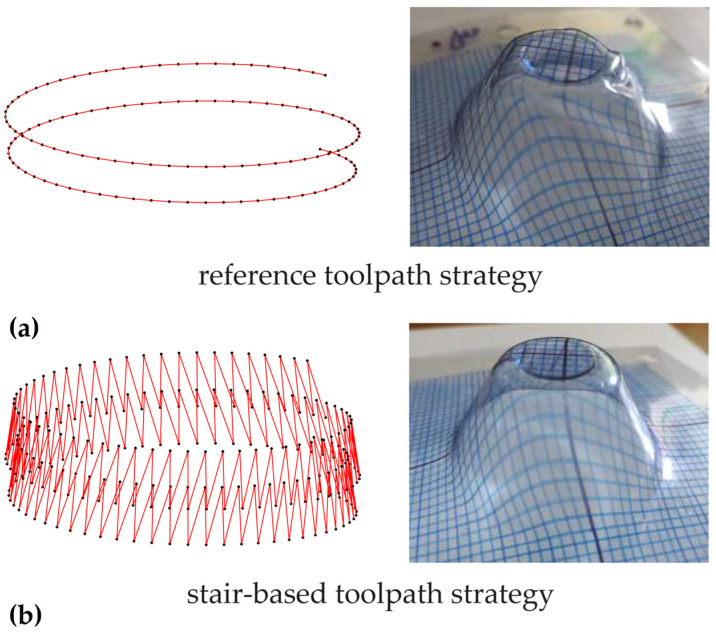
Not-to-scale representation of (**a**) the reference and (**b**) one stair-based toolpath strategy and the corresponding cone frusta.

The stair paths, while resulting in higher working times, help reduce twisting and, more generally, defect phenomena due to a discontinuous and lower torque action; in addition, for these strategies, the influence of the lubrication on surface roughness is quite irrelevant. Figure 6 reports a not-to-scale representation of the reference (Figure 6a) and one stair-based toolpath strategy (Figure 6b), and the corresponding cone frusta.

### 3.2. Preliminary Cold-Rolling

**Figure 7:**
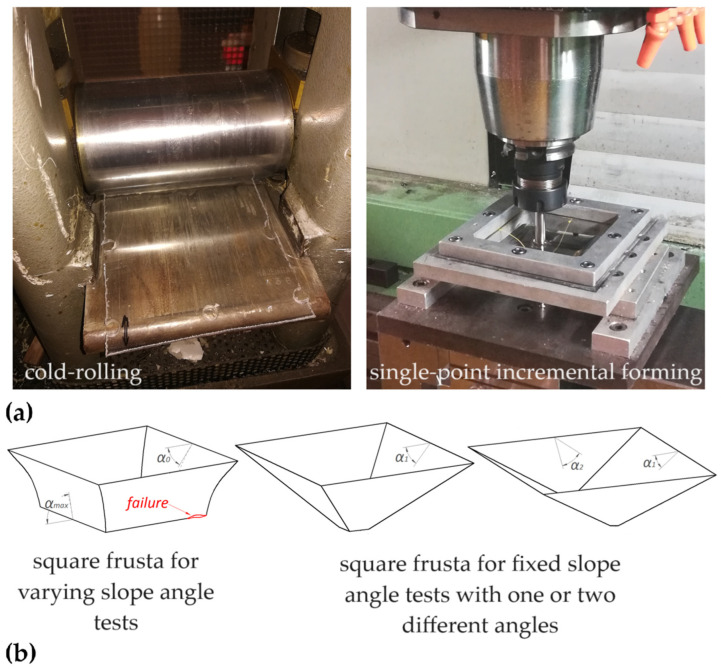
SPIF with preliminary cold-rolling: (**a**) equipment for the processing of the polycarbonate sheets and (**b**) geometries of the square pyramid frusta for the forming tests.

This solution involves a preliminary cold-rolling process and investigates its influence on the SPIF of polycarbonate sheets [75,76]; different thicknesses of the parent sheets (from 1 to 4 mm) and rolling reduction ratios (up to 1/2, along only one or two directions) were considered for the manufacture of square pyramid frusta with varying or fixed slope angles. Figure 7a reports the equipment for processing the polycarbonate sheets and Figure 7b displays the square pyramid frusta for the different forming tests.

Sheet rolling causes work hardening and a decrease in ductility; consequently, the process experiences an increase in forming forces and reduced formability. The effects are more evident with an increase in the reduction ratio, making the sheets very brittle, at a reduction ratio of 1/2. Moreover, due to their anisotropy, a higher risk of failures and defects is observed for sheets rolled in only one direction. This aspect also influences the location and propagation of cracks: the higher forming forces and anisotropy cause wrinkling instability in the sheets rolled in only one direction; on the other hand, twisting is practically the same for both the parent and rolled sheets. Finally, preliminary rolling can be a valid design strategy in some cases; for example, bidirectional rolling can be chosen to obtain components with low slope angles, while unidirectional rolling can be a solution if the components show different slope angles, imposing the rolling reduction along the direction of the shallower faces.

### 3.3. Self-Heating by Tool Rotation and Travelling Speed

**Figure 8:**
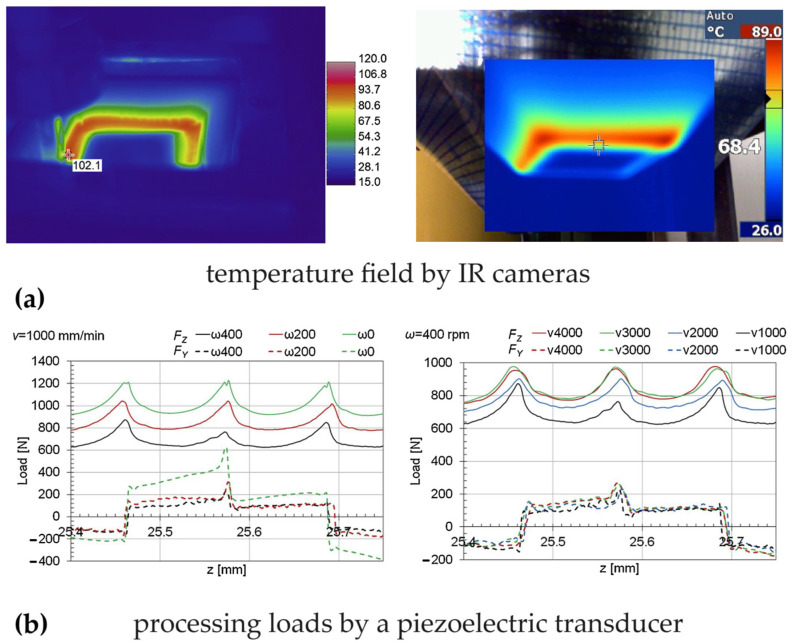
SPIF with self-heating: (**a**) monitoring of the temperature and (**b**) processing loads by varying the tool rotation and the travelling speed.

Other studies [77,78] have examined the self-heating abilities of the polycarbonate sheets during SPIF as a result of the travelling speed and the rotation of the tool. This allows for the control of the temperature at the tool/workpiece contact surface using infrared (IR) cameras (see Figure 8a). The tool rotation (from 0 to 400 rpm) and travelling speed (from 1000 to 4000 mm/min) were varied for the incremental forming of 2 and 3 mm thick sheets and their influence on the temperature and processing loads was determined (see Figure 8b).

These studies show that it is possible to increase the temperature of polycarbonate sheets by appropriately selecting travelling and tool rotation speeds; the latter has the highest influence. Using a tool with a flat end enables a higher frictional heat compared to that with a round-end tool; this allows high temperatures to be reached without excessively high tool rotation speeds.

Higher temperatures (in the range of 10 0 ÷ 120 °C) result in lower processing loads and significantly reduced springback without surface degradation; these temperatures are reached with a rotational speed of 200 rpm and a travelling speed of 1000 mm/min. On the other hand, processing conditions involving temperatures higher than 120 °C cause the opacification of the worked surfaces and a significant deterioration in their quality in terms of roughness; near the glass transition temperature of polycarbonate (≈150 °C), chips are produced from the worked surface.

### 3.4. Contactless Method by Hot Air

**Figure 9:**
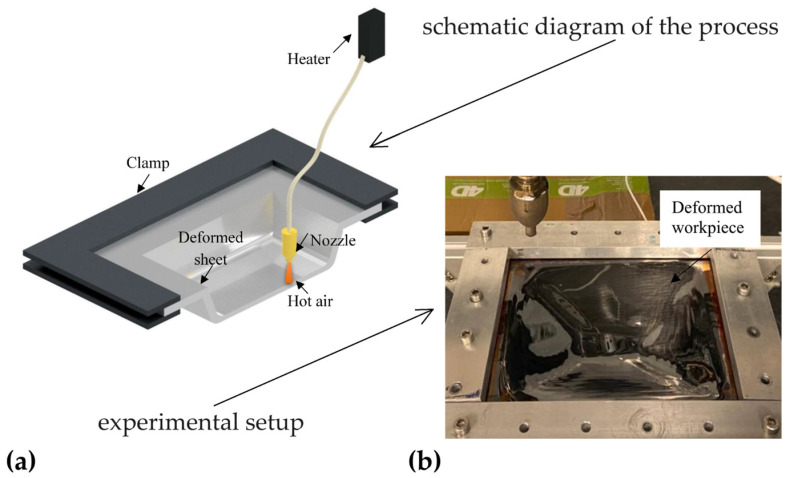
Contactless SPIF process by hot air: (**a**) schematic diagram and (**b**) experimental setup.

In two recent works, Almadani et al. [79,80] propose eliminating the physical interaction between 0.75 mm thick polycarbonate sheets and a rigid forming tool by developing and optimising a contactless SPIF method based on hot compressed air as a deformation tool. The initial studies were carried out at an air temperature of 160 °C, a pressure of 1 bar, and a nozzle speed of 750 mm/min; then, the process’s effectiveness was assessed by using a design of experiments (DOE) with 54 different forming conditions, and the influence of the most significant parameters was evaluated using the response surface method. Figure 9a,b report, respectively, a schematic diagram and the experimental setup after the deformation process.

The studies reveal that the contactless process can be tailored for a wide range of polymer materials; in addition, they highlight the importance of air pressure, air temperature, and feed rate, as these factors influence the process’s formability, surface quality, and variations in profiles and thicknesses.

### 3.5. Numerical Analyses

Since ISF represents one of the novel manufacturing processes, it has gained much attention from researchers and practitioners, leading to the development of several analytical and numerical process models. Consider, for example, artificial intelligence (AI) techniques such as artificial neural networks, genetic algorithms, support vector regression, fuzzy logic, etc., and the finite element method (FEM) [81]. FEM is a numerical modelling technique used to solve a wide range of problems in different fields of engineering and science; it finds approximate solutions to partial differential equations, producing much more detailed results than experimental investigations, often more quickly and less expensively [82]. It can provide visual representations of the results, such as stresses, strains, or temperature fields, which are useful for understanding the behaviour of systems and identifying potential problems or opportunities for improvement.

**Figure 10:**
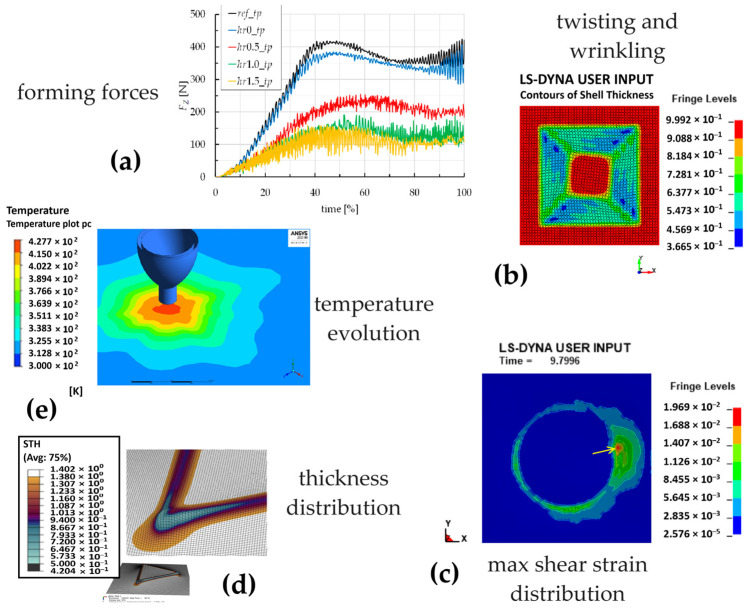
Overview of FEM analyses applied to the incremental forming of polycarbonate sheets: (**a**) trend of the forming forces; (**b**) defects and (**c**) deformation states; (**d**) thickness distribution; (**e**) temperature evolution.

The incremental forming of polymer sheets was frequently analysed using FEM simulations. For example, the axial force in the SPIF of thermoplastic sheets was predicted in [83], while the feasibility of an advanced robotised polymer ISF was investigated in [84]. Regarding the aim of this review, Figure 10 provides an overview of FEM analyses applied to the incremental forming of polycarbonate sheets; they are described in this subsection.

Two authors’ papers aim to identify, through a numerical approach, toolpath strategies for optimising the ISF of polycarbonate [85,86]. The FEM commercial code LS DYNA was used to simulate the process; it is a general-purpose FEM programme used for complex real-world problems related to, among others, the automobile, aerospace, construction, military, manufacturing, and bioengineering industries, whose efficiency was guaranteed by different studies [3,87]. Through an accurate interpretation of the results, FEM analyses represent a powerful tool for process optimisation both directly (in terms of manufacturing time and energy states) and indirectly (by predicting defectiveness and risks of failures through, for example, a careful interpretation of the forming forces; see Figure 10a). Another study using LS DYNA follows a numerical–experimental approach to investigate wrinkling in 1 mm thick polycarbonate sheets (Figure 10b,c), highlighting the critical conditions for the occurrence of this phenomenon and the influence of the toolpath strategy on the deformation mechanisms [88].

Regarding the above-reported strategy of self-heating through the tool rotation and travelling speed, a thermo-mechanical FEM model of the process was developed, calibrated, and validated using an explicit time integration approach within the Abaqus 6.17 framework. It provides accurate predictions of temperature evolution and processing loads [32] and serves as a key tool to predict adverse processing conditions, i.e., process parameter values that lead to excessively high temperatures. The same software and approach were used to determine the stress, strain, and thickness distributions (Figure 10d) during the manufacturing of cone and pyramid frusta (with square and triangular bases) from sheets with two different thicknesses [51], providing significant insight into the process. The FEM predictions show a strong correlation between the sheet thickness distribution and the differences in failures occurring in square and triangular frusta.

Support for experimental works on contactless incremental forming by hot air was provided by numerical analyses using ANSYS 21 Workbench (see the temperature evolution in Figure 10e), employing a computational fluid dynamic (CFD) model [89]. The FEM model can predict the formed part geometries and the strain progression, thereby establishing a solid groundwork for advancing and refining the contactless process.

## 4. Conclusions and Future Perspectives

Although there has been great effort in research on the ISF of metal parts, the process as applied to polymers needs to be studied more deeply. This review, after a concise overview of the process from its origins and the state of the art applied to thermoplastics, focuses on one of the most interesting engineering plastics, polycarbonate. Particular attention is devoted to describing the main characteristics and formability of polycarbonate sheets as a function of the main process parameters; the document then reports some strategies to improve the formability of materials that are difficult to work with at room temperatures using common ISF variants, as well as the accuracy and range of feasible geometries, among other aspects. These solutions range from optimised toolpath strategies to hybrid forming methods.

Regarding technical challenges for the ISF of polycarbonate, and, more generally, thermoplastics, they can encompass several fields, including but not limited to the following:

- More effective analytical mechanics—this allows for a better description of the material behaviour of thermoplastics under ISF conditions;
- The development or improvement of alternative hybrid forming and toolpath strategies—these solutions represent a viable way to improve the process in terms of the material formability and the variety and complexity of the components, among others;
- The increased use of thermo-mechanical numerical simulations—the development of accurate numerical models represents a valid tool to investigate various features, such as deformations and failure mechanisms.

Finally, new research directions are strongly oriented towards aspects involving energy implications, as they are of relevant interest from a sustainable manufacturing perspective. The optimisation of ISF processes represents a viable way towards more efficient and green manufacturing processes [90]; for example, a study on the ISF of polycarbonate and polyvinylchloride, aimed at developing a methodology to appropriately set the process parameters to obtain the best and most cost-effective parts [91], highlighted the ability of ISF to reduce energy consumption compared to conventional processes, resulting in a positive impact on the environment.

## Data Availability

No data available.
